# Sea Hare Hydrolysate-Induced Reduction of Human Non-Small Cell Lung Cancer Cell Growth through Regulation of Macrophage Polarization and Non-Apoptotic Regulated Cell Death Pathways

**DOI:** 10.3390/cancers12030726

**Published:** 2020-03-19

**Authors:** Marie Merci Nyiramana, Soo Buem Cho, Eun-Jin Kim, Min Jun Kim, Ji Hyeon Ryu, Hyun Jae Nam, Nam-Gil Kim, Si-Hyang Park, Yeung Joon Choi, Sang Soo Kang, Myunghwan Jung, Min-Kyoung Shin, Jaehee Han, In-Seok Jang, Dawon Kang

**Affiliations:** 1 Department of Physiology and Institute of Health Sciences, College of Medicine, Gyeongsang National University, Jinju 52727, Korea; mariemerci1994@naver.com (M.M.N.); eunjin1981@hanmail.net (E.-J.K.); wlgus9217@naver.com (J.H.R.); jheehan@gnu.ac.kr (J.H.); 2 Department of Convergence Medical Science, Gyeongsang National University, Jinju 52727, Korea; 3 Department of Radiology, Ewha Womans University Medical Center, Seoul 07804, Korea; kingnose80@gmail.com; 4 Department of Anatomy, College of Medicine, Gyeongsang National University, Jinju 52727, Korea; medic0619@naver.com (M.J.K.); kangss@gnu.ac.kr (S.S.K.); 5 Department of Medicine, College of Medicine, Gyeongsang National University, Jinju 52727, Korea; hjnam98@naver.com; 6 Department of Marine Biology and Aquaculture and Institute of Marine Industry, Gyeongsang National University, Tongyeong 53064, Korea; ngkim@gnu.ac.kr; 7 Sunmarin Biotech, Tongyeong 53064, Korea; hyangi51@hanmail.net; 8 Department of Seafood Science and Technology and Institute of Marine Industry, Gyeongsang National University, Tongyeong 53064, Korea; yjchoi@gnu.ac.kr; 9 Department of Microbiology, College of Medicine, Gyeongsang National University, Jinju 52727, Korea; vetmhjung@gmail.com (M.J.); mkshin@gnu.ac.kr (M.-K.S.); 10 Department of Thoracic and Cardiovascular Surgery, College of Medicine, Gyeongsang National University and Gyeongsang National University Hospital, Jinju 52727, Korea

**Keywords:** immune modulation, lung cancer, macrophage polarization, necroptosis, pyroptosis, sea hare hydrolysates

## Abstract

Sea hare-derived compounds induce macrophage activation and reduce asthmatic parameters in mouse models of allergic asthma. These findings led us to study the role of sea hare hydrolysates (SHH) in cancer pathophysiology. SHH treatment-induced M1 macrophage activation in RAW264.7 cells, peritoneal macrophages, and THP-1 cells, as did lipopolysaccharide (LPS) (+ INF-γ), whereas SHH reduced interleukin (IL)-4 (+IL-13)-induced M2 macrophage polarization. In addition, SHH treatment inhibited the actions of M1 and M2 macrophages, which have anticancer and pro-cancer effects, respectively, in non-small cell lung cancer cells (A549 and HCC-366) and tumor-associated macrophages (TAMs). Furthermore, SHH induced G2/M phase arrest and cell death in A549 cells. SHH also downregulated STAT3 activation in macrophages and A549 cells, and the down-regulation was recovered by colivelin, a STAT3 activator. SHH-induced reduction of M2 polarization and tumor growth was blocked by colivelin treatment. SHH-induced cell death did not occur in the manner of apoptotic signaling pathways, while the death pattern was mediated through pyroptosis/necroptosis, which causes membrane rupture, formation of vacuoles and bleb, activation of caspase-1, and secretion of IL-1β in SHH-treated A549 cells. However, a combination of SHH and colivelin blocked caspase-1 activation. Z-YVAD-FMK and necrostatin-1, pyrotosis and necroptosis inhibitors, attenuated SHH’s effect on the cell viability of A549 cells. Taken together, SHH showed anticancer effects through a cytotoxic effect on A549 cells and a regulatory effect on macrophages in A549 cells. In addition, the SHH-induced anticancer effects were mediated by non-apoptotic regulated cell death pathways under STAT3 inhibition. These results suggest that SHH may be offered as a potential remedy for cancer immunotherapy.

## 1. Introduction

Lung cancer has the highest mortality rate in Korea, and has done so for more than 10 years (Korean Government e-nara index, www.index.go.kr, 28 January 2020). Patients with lung cancer are typically diagnosed at a late stage, after too much progress, because there are no special symptoms at an early stage. Therefore, the methods for lung cancer treatment at the end stage are very limited. Recently, immunotherapy has been considered for patients with end-stage lung cancer. Furthermore, immune anticancer drugs are in the spotlight as a new treatment that compensates the shortcomings of chemotherapy and targeted anticancer drugs. In particular, the contribution of immunotherapeutic agents to non-small cell lung cancer (NSCLC) is increasing [1]. However, despite the benefits of immunotherapy, its use is limited for many lung cancer patients because of the cost and the requirement for treatment standards. The expression levels of programmed cell death ligand 1 (PD-L1) and other relevant biomarkers in NSCLC should be compatible with therapeutic standard [2]. At present, immuno-chemotherapy is attracting attention as a next-generation therapy that helps patients overcome through enhancement of immunity.

Macrophages are a major regulator of inflammatory responses to disease and infections, and a main immune component of the tumor microenvironment [3,4]. Macrophages are polarized into specific phenotypes, generally described as the classically activated macrophage (M1) and the alternatively activated macrophage (M2), depending on the type of stimulant [5,6]. Traditionally, M1 and M2 reflect Type 1 and Type 2 helper T cell (T_h_1 and T_h_2) polarization of immune responses, respectively [5,7], and are associated with inflammatory and anti-inflammatory reactions, respectively [8]. With respect to cancer, macrophage subsets have been found to be highly involved in the cancer microenvironment. M1 exhibits anticancer effects, but M2-polarized tumor-associated macrophages (TAMs) promote tumor growth, progression, angiogenesis, and metastasis in malignant cancers [5,9,10,11]. Developing molecules or modulators that properly regulate macrophage polarization may help to achieve positive results in cancer treatment.

Several therapeutic agents, such as Gefitinib and Imatinib, suppress M2-like polarization in NSCLC [12,13]. Nuclear Factor-kappa B (NF-κB) and/or signal transducer and activator of transcription (STAT) 1 are activated for M1 polarization, while STAT3, STAT6, peroxisome proliferator-activated receptor (PPAR) δ, and/or PPARγ are activated for M2 polarization [14]. Activation of p38 mitogen-activated protein kinase (MAPK) is also involved in M2 polarization [15]. Compared to M1 polarization, signaling pathways regarding M2 polarization are not fully established. The number of molecules identified as inhibitors of M2 polarization is also small.

Sea hares secrete many kinds of bioactive molecules, which have various bioactivities [16,17]. The immunological activities of sea hares have already been demonstrated through our previous studies as follows: sea hare-derived glycosaminoglycans induce macrophage activation [18], and sea hare hydrolysate (SHH), which has higher bioactivities compared to extracts, reduce asthmatic parameters in mouse models of allergic asthma [19]. In addition, sea hare-derived glycosaminoglycans show cytotoxicity in stomach cancer cell AGS, but not in normal mouse ileum epithelial cells (patent KR20110132746A). These findings led us to study the hypothesis that SHH may be able to treat lung cancer through the regulation of macrophages. This study was performed to identify the effects of SHH on macrophage polarization and on growth of NSCLC using mouse and human macrophages, human TAMs, and human NSCLC cells.

## 2. Results

### 2.1. SHH-Induced Polarization of M1 Macrophages in RAW264.7 Cells

**Figure 1:**
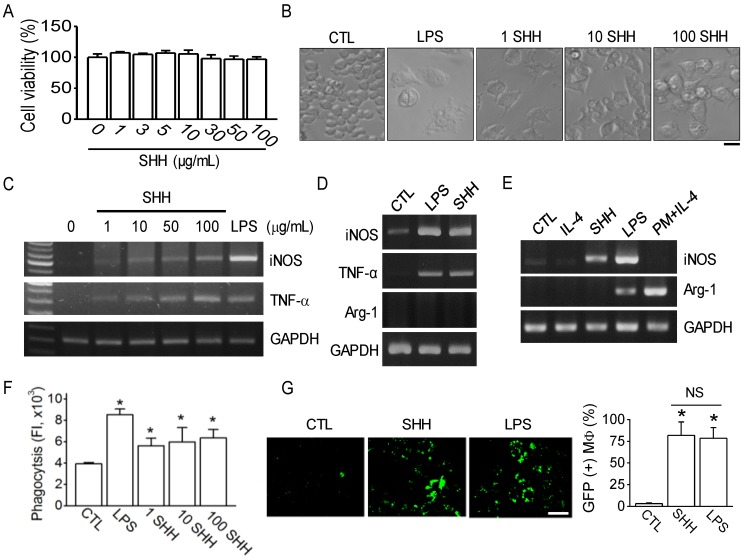
Sea hare hydrolysate-induced polarization to M1 of RAW264.7 cells. (**A**) Effect of sea hare hydrolysate (SHH) on cell viability. Different concentrations of SHH were used to treat RAW264.7 cells for 24 h. Each bar is the mean ± standard deviation (SD) obtained from five independent experiments (*n* = 5). (**B**) Morphological changes in RAW264.7 cells activated by SHH treatment. Numbers (1, 10, and 100) above the figures represent the concentration (μg/mL). LPS (1 μg/mL) was used as a positive control. Scale bar, 15 μm. (**C**) SHH-induced increase in iNOS and TNF-α expression. Glyceraldehyde-3-Phosphate Dehydrogenase (GAPDH) was used as a loading control to compare the mRNA expression level among treatments. (**D**) No expression of Arg-1, a marker of M2, in RAW264.7 cells. LPS (1 μg/mL) was used as a positive control for induction of iNOS and TNF-α expression. (**E**) No effect of IL-4 and SHH on Arg-1 expression in RAW264.7 cells. The Arg-1 was expressed in the IL-4-treated mouse peritoneal macrophages. (**F**) Increase in the phagocytic ability of RAW264.7 cells by SHH treatment. Cells cultured in 96-well black plates were treated with LPS or SHH and loaded with latex bead rabbit IgG FITC complex. The degree of phagocytosis was analyzed using a fluorescence microplate reader. Each bar is the mean ± SD obtained from nine independent experiments (*n* = 9). * *p* < 0.05 compared to control (CTL). FI represents fluorescence intensity. (**G**) RAW264.7 cells phagocytized A549 lung cancer cells. The cancer cells were transfected with green fluorescent protein (GFP) and co-cultured with RAW264.7 cells for 24 h under SHH treatment. Strong green fluorescence instead of dots shows A549 cells transfected with GFP. The bar graph shows the percentages of GFP positive cells (RAW264.7 cells that phagocytized A549 cells). Each bar is the mean ± SD obtained from four independent experiments (*n* = 4). LPS treatment was used as a positive control. NS, not significant. Scale bar, 30 μm. * *p* < 0.05 compared to control (CTL).

The effect of SHH on macrophage polarization was investigated in RAW264.7 cells. To determine the concentration of SHH to be used for subsequent experiments, that does not induce cell death in RAW 264.7 cells, an MTT assay was performed. The cells were cultured in the presence of various concentrations of SHH (1 to 100 μg/mL) for 24 h. The concentrations of SHH tested in this study did not affect the cell viability of RAW264.7 cells (Figure 1A).

To investigate the effect of the concentration on macrophage activation, cells were treated with SHH at three different concentrations (1, 10, and 100 μg/mL). SHH of all concentrations used in this experiment activated RAW264.7 cells. SHH-treated cells showed a large and flat morphology with spreads, vacuoles, and granules compared to the control, as did lipopolysaccharide (LPS) (Figure 1B). The number of cells with vacuoles and granules was larger in the 10 and 100 μg/mL SHH treatments than that in the 1 μg/mL SHH treatment. The cell morphology changed by SHH was similar to the M1 phenotype stimulated by LPS and interferon gamma (IFN-γ) [20].

To identify the M1 polarization state of SHH-treated cells, inducible nitric synthase (iNOS) and tumor necrosis factor (TNF)-α (representative markers for M1 phenotype) mRNA expression patterns were evaluated. SHH treatment increased iNOS and TNF-α mRNA expression in a concentration-dependent manner (Figure 1C). The effect of SHH on iNOS and TNF-α mRNA expression was similar to that of LPS (Figure 1D). Arginase-1 (Arg-1), a marker of the M2 phenotype, was not detected in RAW264.7 cells treated with SHH (100 μg/mL) or LPS (1 μg/mL) (Figure 1D). The response of RAW264.7 cells to interleukin (IL)-4 was also evaluated by detection of Arg-1 expression. RAW264.7 cells did not respond to IL-4 treatment, which typically induces Arg-1 expression in other macrophages. To identify whether Arg-1 is not actually expressed in the RAW264.7 cells under our experimental condition, mouse peritoneal macrophages were adopted. Arg-1 expression, but not iNOS, was detected in the mouse peritoneal macrophage treated with IL-4, indicating that RAW264.7 cells have a strong tendency to polarize into M1 (Figure 1E). SHH-treated cells showed high phagocytic ability, as judged by the in vitro phagocytosis ability assay, which measures the fluorescence intensity of positive cells for fluorescent beads (*p* < 0.05; Figure 1F). The phagocytic ability of SHH-treated RAW264.7 cells was reevaluated by co-culture with RAW264.7 cells and A549 cells transfected with green fluorescent protein (GFP). SHH-activated RAW264.7 cells phagocytized A549 cells, and the phagocytized RAW264.7 cells showed green dots. There was no significant difference between SHH and LPS treatments in the ability of RAW264.7 cells to phagocytize A549 cells (*p* > 0.05; Figure 1G). SHH-untreated RAW264.7 cells showed a very small amount of green fluorescence (Figure 1G).

### 2.2. Reduction of IL-4-Induced M2 Polarization in Mouse Peritoneal Macrophages by SHH 

**Figure 2:**
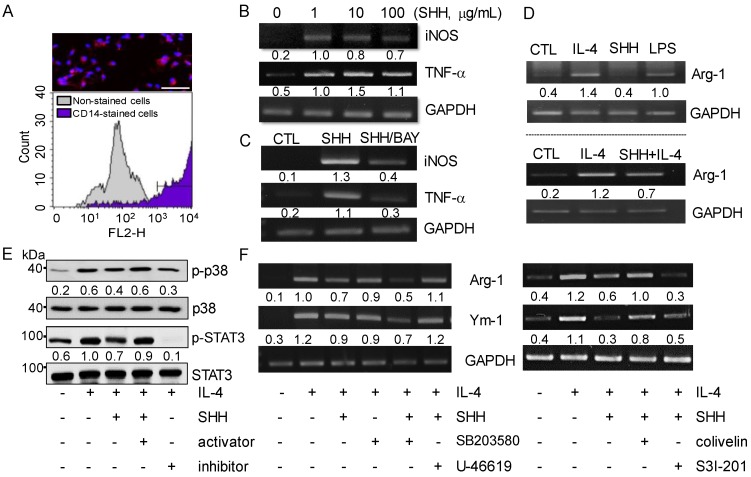
Down-regulation of M2 markers in mouse peritoneal macrophage by SHH treatment. (**A**) Flow cytometry profiles of mouse peritoneal macrophages. CD14 phycoerythrin (PE)-stained attached cells (red, CD14-positive cell; blue, 4′,6-Diamidino-2-phenylindole dihydrochloride (DAPI) for nucleus). Scale bar, 50 μm. (**B**) Increase in iNOS and TNF-α mRNA expression in SHH-treated cells. (**C**) Reduction of SHH-induced upregulation of iNOS and TNF-α by pretreatment with the NF-κB inhibitor. (**D**) IL-4-induced upregulation of Arg-1 reduced by SHH treatment. IL-4 was used as a positive control for the induction of Arg-1. (**E**) Reduction of p38 and STAT3 activities by SHH treatment. IL-4 and SHH were treated simultaneously, and p38 and STAT3 modulators were pretreated for 1 h. The numbers between blots are the normalized ratios of phosphorylated protein to total protein. (**F**) Recovery of SHH-induced decrease in Arg-1 and Ym-1 expression levels by p38 and STAT3 activators. The numbers between gels represent the normalized ratios of the mRNA levels of macrophage markers to that of GAPDH for each lane. Five independent experiments were performed (*n* = 5).

Mouse peritoneal macrophages were used to analyze the expression pattern of the M1 and M2 markers following SHH treatment. Isolated primary macrophages were confirmed by staining with Cluster of differentiation (CD) 14, a marker for monocytes/macrophages. CD14-positive cells stained in red accounted for 89.0 ± 2.0% in the isolated peritoneal cells (Figure 2A). SHH treatment upregulated the expression of iNOS and TNF-α mRNA (Figure 2B). Activation of the NF-κB signaling pathway has been reported to induce M1 macrophage differentiation and the consequent inflammatory effects [14,21]. To establish if NF-κB activation is involved in the SHH-induced upregulation of iNOS and TNF-α mRNA expression, the NF-κB inhibitor BAY11-7085 was combined with SHH treatment. SHH-induced upregulation of iNOS and TNF-α expression was inhibited by pretreatment with BAY11-7085 (Figure 2C), indicating that NF-κB signaling is involved in SHH-induced upregulation of iNOS and TNF-α expression.

The expression of the M2 marker Arg-1 was increased by IL-4 and LPS treatment, but not SHH treatment. Rather, SHH treatment reduced Arg-1 expression upregulated by IL-4 treatment (Figure 2D). We checked whether p38 MAPK and STAT3 signals are related to the SHH effect on M2 polarization. IL-4 activated p38 MAPK and STAT3, but their activation was decreased by SHH treatment in mouse peritoneal macrophages (Figure 2E). The activators (U-46619 for p38 and colivelin for STAT3) combined with SHH recovered the p38 and STAT3 activities inhibited by SHH. In addition, the combination of IL-4 and the specific inhibitors (SB203580 for p38 or S3l-201 for STAT3) showed similar p38 and STAT3 activities to those in combination of IL-4 and SHH (Figure 2E). The IL-4-induced Arg-1 mRNA expression level was highly decreased by SHH treatment. Combined treatment with p38 and STAT3 inhibitors enhanced SHH’s effect on M2 marker mRNA expression. The Ym-1, another M2 marker, expression pattern in response to p38 and STAT3 modulators was similar to the Arg-1 expression pattern (Figure 2F). The expression levels of Arg-1 and Ym-1 reduced by SHH were also recovered by combination with activators (U46619 and colivelin).

### 2.3. SHH-Induced Suppression of M2 Macrophage Polarization in Human Tumor-Associated Macrophages (TAMs)

**Figure 3:**
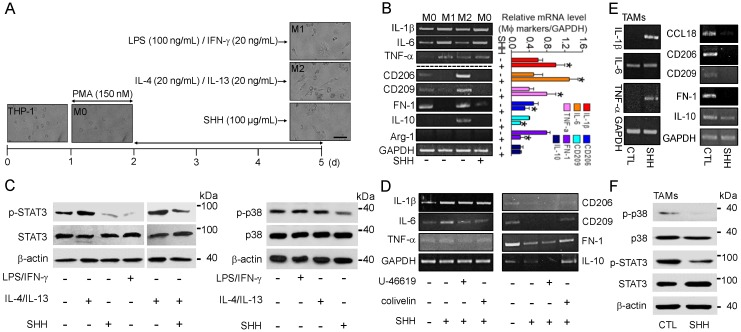
SHH-induced upregulation of M1 markers and downregulation of M2 markers in both human macrophages and tumor-associated macrophages (TAMs). (**A**) PMA-treated THP-1 polarization into M1 and M2 macrophages by LPS/IFN-γ and IL-4/IL-13 treatment for 3 days, respectively. M0 macrophages were activated by SHH. Ten independent experiments were performed (*n* = 10). Scale bar, 50 μm. (**B**) SHH-induced upregulation of IL-1β, IL-6, and TNF-α and downregulation of CD206, CD209, and fibronectin-1 (FN-1) in the macrophages. SHH was added to M0 cells for 3 days. The bar graph shows the relative mRNA levels of the macrophage markers compared to that of GAPDH. Each bar is the mean ± SD obtained from four independent experiments (*n* = 4). * *p* < 0.05 compared to each corresponding control. (**C**) SHH-induced downregulation of STAT3 and p38 activities in THP-1-differentiated M0 cells. LPS/IFN-γ, IL-4/IL-13, and/or SHH was added to M0 cells for 8 h. Total protein (35 μg of protein per lane) was analyzed by immunoblotting assay. (**D**) Reduction of SHH’s effect on the mRNA expression of the M1 and M2 markers by STAT3 activator. U-46619, colivelin, and SHH were added to M0 cells for 48 h. (**E**) SHH-induced upregulation of IL-1β, IL-6, and TNF-α and downregulation of CD206, CD209, fibronectin-1 (FN-1), and IL-10 in TAMs. SHH was added to TAMs for 48 h. (**F**) SHH-induced downregulation of STAT3 and p38 activities in TAMs. SHH was added to TAMs for 8 h. Total protein (35 μg of protein per lane) was analyzed by immunoblotting assay. GAPDH and β-actin were used as loading controls for comparing the mRNA and protein expression levels among treatments, respectively. Five independent experiments were performed (*n* = 5).

SHH treatment demonstrated an induction of M1 polarization and a reduction of M2 polarization in the mouse macrophage cell line and primary cells (Figure 1 and Figure 2). The role of SHH in human macrophages was further studied. Tamm-Horsfall protein-1 (THP-1) cells, a human monocytic cell line, were differentiated to macrophages (M0) by phorbol-12-myristate-13-acetate (PMA; 150 nM) treatment. The M0 cells were further polarized to M1 cells by treatment with LPS (100 ng/mL) and IFN-γ (20 ng/mL) and to M2 cells by treatment with IL-4 (20 ng/mL) and IL-13 (20 ng/mL) for 3 days. SHH (100 μg/mL ) added to the M0 cells at the time of the stimulant’s treatment for polarization. Changes in the morphology and expression levels of the M1 and M2 markers were observed in M0 cells treated with inducers for M1 and M2 polarization or SHH. Most of the LPS/IFN-γ-treated cells exhibited the M1 phenotype with short spreading, vacuoles, and flat round shape, while many IL-4/IL-13-treated cells exhibited an elongated shape of the M2 phenotype. SHH-treated cells showed a similar phenotype to M1 rather than M2 (Figure 3A).

As shown in Figure 3B, SHH treatment increased the expression of M1 markers (IL-1β, IL-6, and TNF-α), whereas it reduced the expression of M2 markers (CD206, CD209, and fibronectin-1). IL-10 showed very low expression in M0, so that it was difficult to compare its changes in cells treated with SHH. The Arg-1 M2 marker was not detected in human macrophages, despite numerous trials (Figure 3B), like previous studies showed [22,23].

The p38 MAPK and STAT3 activities were investigated in M0 cells cultured for 8 h with LPS/IFN-γ, IL-4/IL-13, and/or SHH. SHH treatment markedly reduced p38 and STAT3 activities in M0 cells, like LPS/IFN-γ did. The STAT3 activity was high in the IL-4/IL-13 treatment, whereas it was low in the LPS/IFN-γ-treated cells. Moreover, SHH dramatically decreased IL-4/IL-13-induced STAT3 activity (Figure 3C). The activators of p38 and STAT3 were pretreated before SHH treatment of the M0 cells. A downregulation of M2 markers and an upregulation of the M1 markers by SHH were inhibited by the STAT3 activator colivelin, but not the p38 activator U46619 in the downregulation of M2 markers (Figure 3D). TAMs were produced to identify SHH’s effect on tissue-resident macrophages present in the tumor microenvironment. M0 cells were cocultured with A549 cells at a 1:1 ratio for 4 days to establish a TAM-like phenotype, with and without SHH treatment, as previously described [24]. The cells were washed at the end of the incubation to remove the remaining cancer cells from the culture dish. RNA was extracted from TAMs, and semi-quantitative polymerase chain reaction (PCR) was used to measure M1 and M2 marker expression. TAMs highly expressed M2 markers compared to M1 markers, indicating that TAMs exhibit an M2-like phenotype. SHH reduced the expression levels of M2 markers (CCL18, CD206, CD209, fibronectin-1, and IL-10), whereas it increased the expression of M1 markers (IL-1β, IL-6, and TNF-α) (Figure 3E). In TAMs, STAT3 and p38 activities were markedly reduced by SHH treatment (Figure 3F).

### 2.4. Anti-Cancer Effect of SHH by Modulation of Macrophages

**Figure 4:**
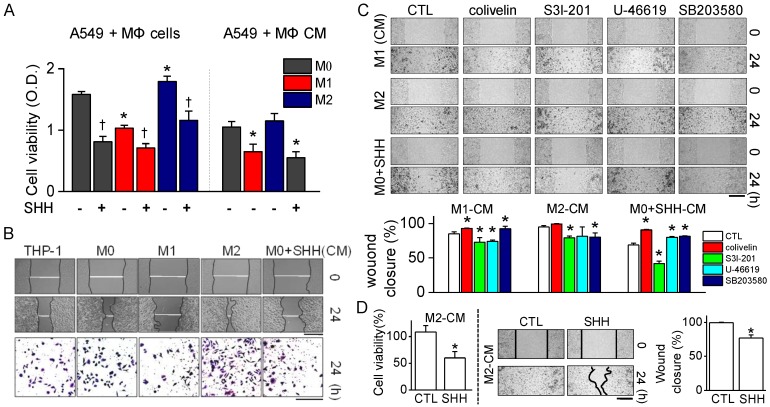
Proliferation and migration of A549 cells reduced by SHH treatment. (**A**) Effect of SHH on cell viability of A549 cells cultured with different types of macrophages or treated with molecules secreted from macrophages. Each bar is the mean ± SD obtained from eight independent experiments (*n* = 8). * *p* < 0.05 compared to M0 co-culture. ^†^ *p* < 0.05 compared to each corresponding control (without SHH). (**B**) Effect of SHH on wound healing. Phase-contrast images of wound areas at 0 and 24 h after wounding of A549 cells. Bottom panel shows migration ability of A549 cells treated with each macrophage conditioned medium (CM). The CMs were from the culture media of THP-1, M0, M1, M2, and SHH-treated M0 cells. Migrated cells remaining on the bottom surface were stained with 0.5% methylene blue. Scale bars, 500 μm for wound closure and 100 μm for migration. (**C**) Effect of STAT3 and p38 modulators on wound healing reduced by SHH. The cells were treated with CMs from M1, M2, and SHH-treated M0 cells for 24 h. Scale bar, 100 μm. Bar graphs show percentage of wound closure in the cells treated with the indicated chemicals. Five independent experiments were performed (*n* = 5). * *p* < 0.05 compared to each corresponding control. (**D**) Effect of SHH on cell viability and wound healing increased by M2-CM. SHH were added to A549 cells for 24 h in the presence of M2-CM. Scale bar, 100 μm. Data are shown as the mean ± SD of four independent experiments (*n* = 4). * *p* < 0.05 compared to the control.

To identify whether SHH control the effect of M1 and M2 cells on the growth of cancer cells, the macrophages were co-cultured with A549 cells at a 5:1 ratio for 48 h. Co-culture with M1 cells significantly decreased the cell viability of A549 cells, while coculture with M2 increased the cell viability compared to that in coculture with M0. SHH treatment induced growth inhibition in all groups (*p* < 0.05; Figure 4A).

SHH only treatment did not affect M0 cell viability, indicating that SHH affect the viability of A549 cells. In addition, the molecules secreted from M1 and M2 cells (supernatants, conditioned medium (CM)) also induced a decrease and an increase in A549 cell viability, respectively, compared to M0-CM (*p* < 0.05; Figure 4A). The macrophage CM affected the proliferation and migration of A549 cells. M1-CM reduced A549 cell proliferation and migration, whereas M2-CM increased them. SHH-treated CM dramatically reduced the cell proliferation and migration of A549 cells, like M1-CM did (Figure 4B). Cells treated with M0-CM showed a similar effect on proliferation and migration of A549 cells compared to the control (THP-1-CM). The three-dimensional (3D) migration assay also showed a decrease and an increase in the treatment with M1-CM and M2-CM, respectively (Figure 4B, bottom panel).

The involvement of p38 and STAT3 in proliferation and migration was checked. A combination of M1-CM or SHH and activators (U46619 for p38 and colivelin for STAT3) enhanced cell proliferation and migration, whereas a combination with S3l-201, an inhibitor of STAT3, decreased them, compared to M1-CM or SHH-CM only treatment. However, the p38 inhibitor SB203580 greatly increased cell proliferation and migration. A combination of M2-CM and colivelin increased cell proliferation and migration, whereas a combination of S3l-201 decreased them. Both U46619 and SB203580 reduced cell proliferation and migration, compared to M2-CM only treatment. Colivelin treatment significantly inhibited SHH’s effect on wound healing, whereas S3l-201 increased its effect (*p* < 0.05; Figure 4C). However, in cell proliferation and migration, both U46619 and SB203580 reduced the effect of SHH. The M2-CM-induced increase in cell viability and wound healing was significantly reduced by the SHH combination (*p* < 0.05; Figure 4D). These results show that STAT3 was primarily involved in the SHH-induced reduction in the cell proliferation and migration of A549 cells.

### 2.5. Cytotoxic Effect of SHH on A549 Cells

**Figure 5:**
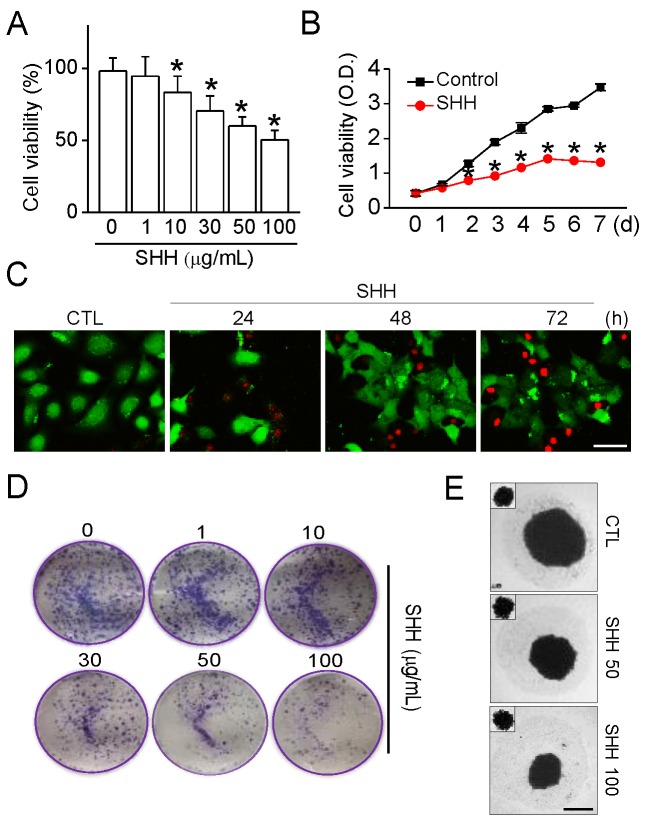
SHH-induced inhibition of A549 cell growth. (**A**) concentration-dependent effect of SHH on cell viability. Cells were exposed to SHH (1, 10, 30, 50, or 100 μg/mL) for 48 h. Each bar is the mean ± SD obtained from seven independent experiments (*n* = 7). * *p* < 0.05 compared to the control. (**B**) Cell growth reduced by SHH treatment in a time-dependent manner. A549 cells were cultured in the presence or absence of SHH (100 μg/mL) for 7 days. The cell viability was analyzed at the indicated time. Each data is the mean ± SD obtained from eight independent experiments (*n* = 8). * *p* < 0.05 compared to each corresponding control. (**C**) SHH-induced cell death. Time-dependent increase in the number of dead cells. Live and dead cells are displayed in green (carcein) and red (propidium iodide, PI), respectively. Scale bar, 50 μm. (**D**,**E**) Colony formation (**D**) and spheroid growth (**E**) of A549 cells inhibited by SHH. Cells were exposed to different concentrations of SHH for 2 days in the colony formation assay and for 10 days in the spheroid growth assay. In the spheroid formation assay, the small square in the upper left coner shows the size of speroids on day 4 of culture before SHH treatment. Scale bar, 50 μm. Four independent experiments were performed (**C**,**D**,**E**; *n* = 4).

An MTT assay was performed to assess whether SHH exhibit cytotoxicity on A549 cells. Different concentrations (1 to 100 μg/mL) of SHH were added to A549 cells for 48 h. SHH treatment significantly decreased the cell viability of A549 cells in a concentration-dependent manner (*p* < 0.05; Figure 5A). Treatments with 10, 30, 50, and 100 μg/mL SHH reduced cell viability by 16.6%, 29.6%, 39.9%, and 49.5%, respectively (Figure 5A). A low concentration of SHH (1 μg/mL) did not affect cell viability. In subsequent experiments, the 100 μg/mL SHH that inhibits approximately 50% of cell viability was used. The SHH was added to A549 cells for 7 days. SHH reduced the growth of A549 cells compared to the control in a time-dependent manner. The gap in growth between the control and the SHH treatment increased over time (*p* < 0.05; Figure 5B). There was also an increase in the number of propium iodide (PI)-positive dead cells in the long-term incubation of SHH (Figure 5C). In addition, the soft agar colony formation and tumor spheroid assays showed that SHH had concentration-dependent tumor suppressive effects on A549 cells (Figure 5D,E).

**Figure 6:**
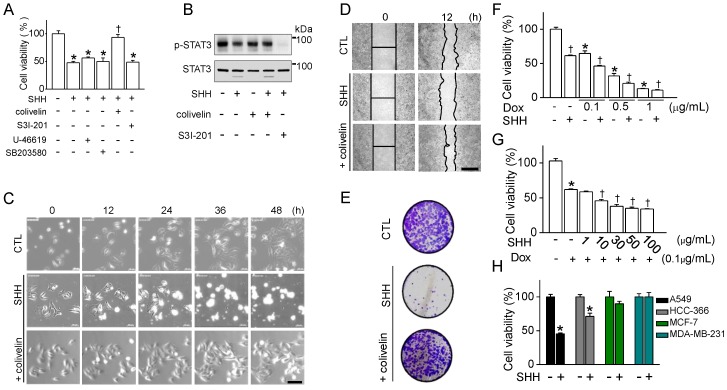
Anti-cancer effect of SHH on A549 cells through inhibition of STAT3 activity. (**A**) Effect of the p38 and STAT3 modulators on SHH-induced cell death. Cells were exposed to SHH (100 μg/mL) for 48 h, and the modulators were pretreated for 1 h. Each bar represents the mean ± SD of four independent experiments (*n* = 4). * *p* < 0.05 compared to the control (no treatment). ^†^ *p* < 0.05 compared to SHH only treatment. (**B**) SHH-induced downregulation of STAT3 activity. SHH was added to A549 cells for 8 h. Total protein (35 μg of protein per lane) was analyzed by immunoblotting assay. (**C**) Real-time images of A549 cells were recorded for 48 h with and without colivelin. (**D**) Effect of colivelin on SHH-induced inhibition of wound healing. Phase-contrast images of wound areas at 0 and 12 h after wounding of A549 cells. (**E**) Reduction of SHH-induced decrease in colony formation of A549 cells by colivelin. Cells were exposed to SHH and colivelin for 48 h. After washing off the chemicals, the cells were further cultured for 10 days for colonization. (**F**,**G**) Synergistic effect of SHH on doxorubicin-induced cell death. Cells were treated with the indicated concentrations of doxorubicin and/or SHH for 48 h. Different concentrations of doxorubicin (**F**) and SHH (**G**). (**H**) Different effects of SHH between non-small cell lung cancer (NSCLC) and breast cancer cells. The cells were exposed to SHH for 48 h. Each bar represents the mean ± SD of four independent experiments (*n* = 4). * *p* < 0.05 compared to no treatment with chemicals. ^†^ *p* < 0.05 compared to each corresponding control.

To identify the involvement of p38 MAPK and STAT3 in the suppression of the cancer cell growth caused by SHH, p38 and STAT3 activators were added to A549 cells prior to SHH treatment. Colivelin pretreatment significantly restored the cell viability reduced by SHH treatment, whereas U46619 did not inhibit SHH-induced cell death (*p* < 0.05; Figure 6A). SHH decreased the activity of STAT3 in A549 cells. The combination of colivelin blocked the inhibition of STAT3, while the combination of S3l-201 reduced STAT3 activity dramatically (Figure 6B). Real-time images showed that treatment combined with colivelin reduced SHH’s lytic cell death (Figure 6C). Wound healing was also restored when SHH and colivelin were combined (Figure 6D). The colony formation assay also showed that colivelin cotreatment prevented SHH-induced reduction of A549 cell proliferation (Figure 6E). Doxorubicin used for the treatment of lung cancer and SHH were co-added to A549 cells for 48 h in order to evaluate the effect of SHH for adjuvant chemotherapy. Doxorubicin significantly caused concentration-dependent cell death. A combination of SHH and doxorubicin significantly enhanced the effect of doxorubicin on cell death (*p* < 0.05; Figure 6F). A low concentration of doxorubicin (0.1 μg/mL) was combined with various concentrations of SHH. As the SHH concentration rose, there was a significant decrease in the viability of A549 cells (*p* < 0.05; Figure 6G). SHH significantly inhibited the cell viability of NSCLC (A549 and HCC-336), but not breast cancer cells (MCF-7 and MDA-MB-231) (*p* < 0.05; Figure 6H).

### 2.6. SHH-Induced Growth Inhibition of A549 Cells

**Figure 7:**
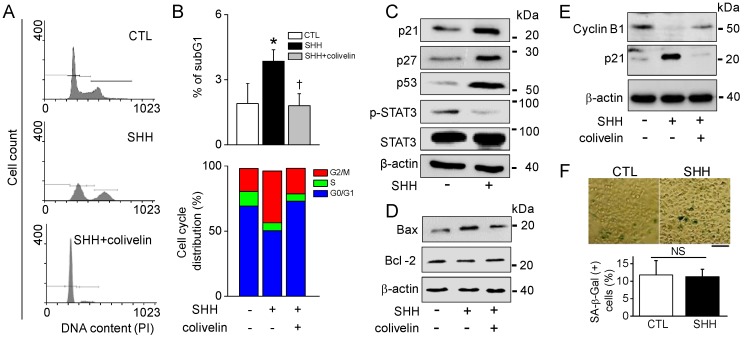
SHH-induced G2/M cell cycle arrest. (**A**,**B**) G2/M cell cycle arrest increased in the SHH treatment. A combination of SHH and colivelin reduced G2/M cell cycle arrest. The apoptosis and cell cycle arrest were quantified by fluorescence-activated cell sorting (FACS) analysis with PI in A549 cells treated with SHH for 48 h. * *p* < 0.05 compared to the control. ^†^ *p* < 0.05 compared to the SHH treatment. Each bar is the mean ± SD obtained from five independent experiments (*n* = 5). (**C**–**E**) Western blot analyses for cell cycle arrest-related proteins. Total protein was obtained from cells in the presence or absence of SHH for 48 h. Colivelin was pretreated 1 h before SHH treatment. Four independent experiments were performed (**C**,**D**,**E**; *n* = 4). (**F**) Representative SA-β-Gal staining images. SA-β-Gal-positive cells show blue color. A549 cells were exposed to SHH for 48 h. Scale bar, 200 μm. The bar graph shows the percentage of SA-β-Gal-positive cells. Each bar is the mean ± SD obtained from four independent experiments (*n* = 4). NS, not significant.

To explore the underlying mechanism by which SHH suppresses A549 cell growth, the distribution of the cell cycle phase in A549 cells was examined through flow cytometric analysis with PI as a staining agent. Treatment with SHH for 48 h resulted in a dramatic phase arrest of G2/M, whereas the number of cells in phase S and G0/G1 was markedly reduced (Figure 7A). As shown in Figure 7A, G2/M arrest was restored by the treatment combined with colivelin. SHH treatment increased the number of cells showing sub-G1, but the sub-G1 population decreased in the combination of SHH and colivelin. The cell fraction showing G2/M arrest in SHH treatment was significantly increased by two-fold (17.7% in control vs. 39.9% in SHH). Colivelin also restored the G2/M arrest (*p* < 0.05; Figure 7B).

Cell cycle arrest-related protein levels were determined in SHH-treated cells. The level of expression of p-STAT3 was decreased, while the level of expressions of p21, p27, and p53 were upregulated in A549 cells treated with SHH (Figure 7C). Furthermore, pro-apoptotic Bax and anti-apoptotic Bcl-2 were upregulated and downregulated, respectively, in SHH-treated cells. Their expression levels in the presence of colivelin were restored close to the level of control (Figure 7D). The level of expression of G2/mitotic–specific cyclinB1 was decreased in the SHH-treated cells, but the level of cyclinB1 expression was restored in the combination of SHH and colivelin (Figure 7E). The senescence-associated β-galactosidase (SA-β-Gal) assay was performed to identify whether SHH induce cellular senescence. There were no significant changes in the number of SA-β-Gal positive cells between control and SHH treatment (*p* > 0.05; Figure 7F).

### 2.7. SHH-Induced Cell Death through Non-Apoptotic Regulated Cell Death Pathway

**Figure 8:**
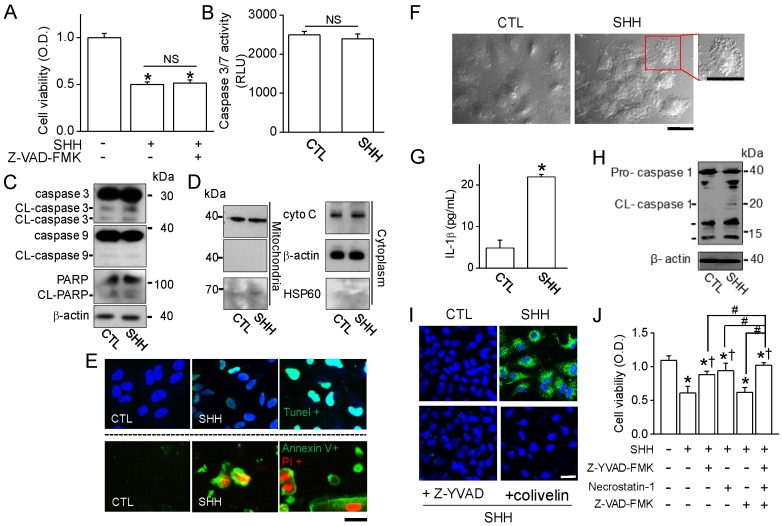
SHH-induced cell death through complex death pathways. (**A**) No effect of Z-VAD-FMK (a pan caspase inhibitor) on A549 cell viability. Cells were exposed to SHH for 48 h, and Z-VAD-FMK was added 1 h before SHH treatment. * *p* < 0.05 compared to the control. NS, not significant. (**B**) No activation of caspase 3/7 by SHH treatment. Luminescence was measured and represented as relative luminescence unit (RLU). (**C**) No cleavage of caspase 3, caspase 9, or PARP by SHH. Cells were treated with SHH for 48 h. A Western blot analysis was performed with the indicated antibodies (against caspase-3, caspase-9, and PARP, and β-tubulin). (**D**) No changes in cytochrome C (cyto C) expression pattern by SHH treatment. Cells were treated with SHH for 48 h and fractionated into mitochondrial and cytosolic components. Fractions were analyzed to assess the release of cyto C. An aliquot of total cell lysate (30 μg of protein per lane) was analyzed by immunoblotting. (**E**) TUNEL and Annexin V/PI staining in SHH-treated cells. Representative photomicrographs of A549 cells labeled with TUNEL fluorescent dye (upper panel, TUNEL in green and DAPI in blue) and Annexin V/PI images (bottom panel, Annexin V in green and PI in red) after exposure of the cells to SHH for 48 h. Scale bar, 30 µm. (**F**) Morphological changes in SHH-treated cells. Representative vacuolization of A549 cells is shown in the expanded image. Scale bar, 30 µm. (**G**) SHH-induced increase in IL-1β secretion. * *p* < 0.05 compared to the control. (**H**,**I**) Activation of caspase 1 by SHH treatment. The activation of caspase-1 was detected by Western blotting (**H**) and immunocytochemistry (**I**) assays. Z-YVAD-FMK and colivelin were added to the cells 1 h before SHH treatment. Scale bar, 30 µm. (**J**) Effect of the inhibitors of pyroptosis and necroptosis on SHH-induced cell death. Cells were pretreated with Z-YVAD-FMK, necrostatin-1, and Z-VAD-FMK for 1 h before SHH treatment for 48 h. Cell viability was determined by an MTT assay. * *p* < 0.05 compared to the control (no treatment). ^†^ *p* < 0.05 compared to the SHH treatment. Each bar is the mean ± SD obtained from five independent experiments (*n* = 5). Four independent experiments were performed (**C**,**D**,**E**,**H**,**I**; *n* = 4). NS, not significant.

SHH induced growth inhibition and cell death. However, the percentage of apoptotic cells was not high compared to other chemicals, as judged by fluorescence-activated cell sorting (FACS) analysis (Figure 7A,B). The mechanisms of SHH-induced cell death in A549 cells were studied. First of all, apoptotic signals were analyzed using immunoblotting assay. Apoptotic signals were not observed in A549 cells treated with SHH for 48 h; no recovery of SHH-induced cell death by pretreatment with a pan-caspase inhibitor (20 μM Z-VAD-FMK; Figure 8A), no changes in caspase-3/7 activity (Figure 8B), no cleavage of caspase-3/-9 and PARP (*n* = 5; Figure 8C), and no changes in cytochrome C expression pattern were observed (Figure 8D). In addition, SHH had no effect on changes in intracellular Ca^2+^ levels, mitochondrial membrane potential (MMP), or reactive oxygen species (ROS) generation. However, TUNEL-positive cells were seen in SHH-treated cells (Figure 8E, upper panel). In addition, most of the cells treated with SHH for 48 h showed double positive for Annexin V-FITC and PI (Figure 8E, lower panel), indicating that SHH induces cell membrane alteration in A549 cells.

SHH-treated cells showed dramatic morphological changes; namely, an increase in cytoplasmic granulation, vacuolization, and membrane blebbing (Figure 8F). These morphological changes were similar to those in necroptotic and pyroptotic cells, which are two pathways of necrotic cell death. They are characterized by plasma membrane rupture, and the pyroptotic and necroptotic membrane damages are related to IL-1β release. SHH induced an increase in the secretion of IL-1β release in A549 cells (Figure 8G). Caspase-1 activity was dramatically increased by SHH treatment (Figure 8H,I), while the activity was decreased in A549 cells treated with Z-YVAD-FMK (a caspase-1 inhibitor) or colivelin (a STAT3 activator) (Figure 8I). The effect of pyroptosis-and necroptosis-related chemicals were evaluated on SHH-induced cell death. Z-YVAD-FMK (10 μM) and/or necrostatin-1 (a necroptosis inhibitor, 1 μM) were added to the A549 cells 2 h prior to SHH treatment. A combination of SHH and Z-YVAD-FMK or necrostatin-1 was significantly attenuated in SHH-induced cell death by approximately 50% (44.1–53.3%), but the recovery level was not as high as the control (*p* < 0.05; Figure 8J). Complex treatments (Z-YVAD-FMK + necrostatin-1) significantly enhanced the effect of Z-YVAD-FMK or necrostatin on the recovery of SHH-induced cell death (*p* < 0.05). Z-VAD-FMK had no additional effect on the recovery of SHH-induced cell death.

**Figure 9:**
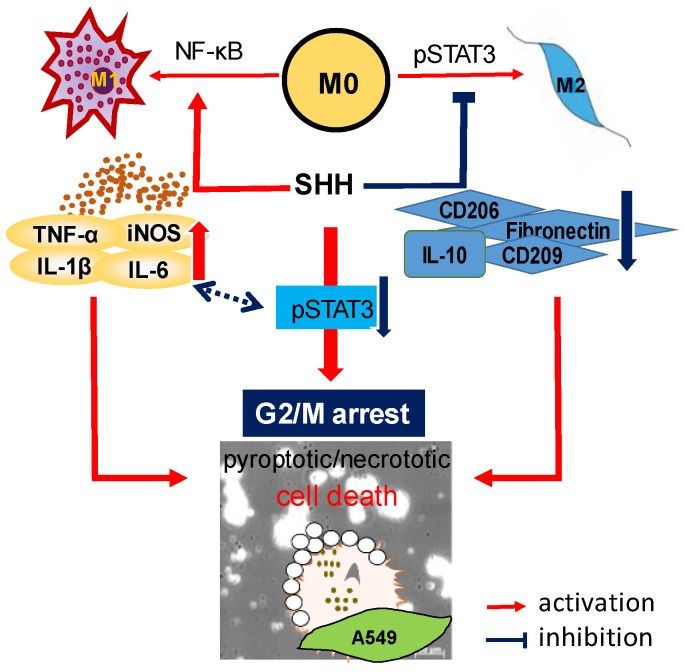
Schematic representation of SHH-induced cell death and inhibition of cell growth of A549. SHH inhibits STAT3 activation, which blocks the activation of p53 and its downstream activation of p21 and p27, and the inhibition of cyclin B1. Finally, G2/M phase arrest occurred. SHH treatment induces M1 activation and M2 suppression, which cause cancer cell death, through molecules secreted from macrophages. SHH-induced cell death is caused by IL-1β-mediated cell death mechanisms, which is preceded by caspase-1 activation. The blue two-way dotted arrows represent each other’s crosstalk. Pro-inflammatory cytokines may induce suppression of pSTAT3, and low pSTAT3 activity may induces M1 activation, which increases the secretion of pro-inflammatory cytokines.

The overall results of this study are summarized in Figure 9.

## 3. Discussion

TAMs, which are abundant in most types of malignant tumors, show an M2 profile. In this study, in TAMs, the expression levels of M2 markers were high (Figure 3E). High infiltrations of M2 in tumor islets and stroma are associated with reduced overall survival in NSCLC [25]. NSCLCs are relatively insensitive to radiotherapy and/or chemotherapy compared to small cell lung cancers (SCLCs) [26]. The development of anti-cancer agents that reduce M2 function is needed to treat NSCLCs with drug resistance and malignant tendencies.

SHH have many benefits for treating lung cancer, namely, M1 activation, M2 suppression, growth inhibition, migration inhibition, and cytotoxicity in cancer cells. This study demonstrates the effects of SHH on the regulating macrophages in mouse macrophage cell line RAW264.7 cells, mouse primary peritoneal macrophages, a human monocytic cell line THP-1 cell, and human TAMs. M1 markers (iNOS and TNF-α) were upregulated and M2 markers (Arg-1 and Ym-1) were downregulated in mouse macrophages. In human macrophages (THP-1-differentiated macrophages), similary, SHH induced an increase in M1 markers (IL-1β, IL-6, and TNF-α) and a decrease in M2 markers (CD206, CD209, fibronectin-1, and IL-10). However, the expression pattern of macrophage markers differed by species and cell type. The mouse M2 marker Arg-1 was not detected in human THP-1-differentiated M2. SHH intrincically modulated the expression of macrophage markers in human macrophages, but not in mouse macrophages. On the other hand, SHH reduced the expression level of the M2 markers upregulated by IL-4 in mouse primary peritoneal macrophages. RAW264.7 cells predominantly show an M1 phenotype, which does not respond to IL-4 treatment (Figure 1E). As a result, SHH have a regulatory effect on both mouse and human macrophages.

The anticancer effects of SHH on cell viability, colony formation, and cell cycle distribution were investigated in A549 cells. The effects were compared to macrophage conditioned medium and doxorubicin. In line with earlier studies, M1 reduced the cell viability and wound healing of A549 cells, whereas M2 enhanced them (Figure 4). SHH reduced the cell viability and wound healing of A549 cells, like M1 did. The anticancer effect of SHH may be due to M1 activation, which shows high phagocytic ability toward cancer cells, oncolytic ability by protease secretion and inflammasome activation. SHH-activated M1 produced pro-inflammatory mediators such as IL-1β, IL-6, TNF-α, and NO. In addition, other molecules are also secreted from activated macrophages. These molecules, including pro-inflammatory cytokines, have the potential to induce lysis in cancer cells through STAT3 inhibition. SHH induces NF-κB activation for induction of M1. Activated STAT3 and NF-κB crosstalk to promote cancer progression [27]. Activation of NF-κB by SHH may reduce the anticancer effect of SHH itself. Interestingly, however, SHH induces activation of NF-κB and inhibition of STAT3 in macrophages and NSCLC cells. As a result, various inflammatory cytokines secreted from M1 activated by SHH are likely to be involved in the anticancer effect of SHH through inhibition of STAT3 activation.

The cell viability and wound healing were regulated by p38 and STAT3 modulators; thus, p38 and STAT3 are promising targets for chemotherapy. Persistent phosphorylation of p38 and STAT3 has been reported in NSCLC. The constitutive activation of p38 and STAT3 is related to increase in cell proliferation and metastasis in NSCLC [28,29,30], and inhibition of p38 and STAT3 reduces the metastatic potential of NSCLC [30,31]. In this study, however, there was no significant difference between the p38 activator and inhibitor treatments in the proliferation and migration of cancer cells. On the other hand, STAT3 activator recovered wound healing reduced by SHH, and its inhibitor enhanced SHH’s effect (Figure 4). These results indicate that SHH’s anticancer effects are strongly mediated by low STAT3 activity.

In addition, the anticancer effect of SHH may be due to M2 suppression, which reduces tumor immunosurveillance escape, angiogenesis, and matrix remodelin, is also contributed. p38 inhbition downregulates the activation of M2 markers in mouse peritoneal macrophages [15] and the human monocytic cell line U937 [32]. STAT3 signaling polarizes macrophages toward M2 [33], and inhibition of the IL-6/STAT3 signaling pathway turns macrophages into M1 [34]. In line with previous studies, p38 and STAT3 were involved in the regulation of mouse and human macrophage polarization. The expression levels of the M2 markers downregulated by SHH were restored by p38 and STAT3 activators in mouse, but not in human macrophages. In human macrophages, only STAT3 activator functions on M2 markers reduced by SHH. SHH reduced STAT3 activity in A549 cells, and the combination of the STAT3 activator and SHH reduced the effect of SHH on STAT3 phosphorylation, cell viability, wound healing, cell growth, colony formation, G2/M phase arrest, and caspase-1 activation (Figure 4, Figure 5, Figure 6, Figure 7 and Figure 8). These results indicate that STAT3 is highly involved in SHH-induced suppression of human M2 and cancer cells.

SHH enhanced the cytotoxic effects of doxorubicin in A549 cells. The cytotoxic effect of SHH was more specific to NSCLC (A549 and HCC366) compared to breast cancer cells (MCF-7 and MDA-MB-231) (Figure 6). In some NSCLC cells, blockade of STAT3 induces apoptois with the induction of Bax and the reduction of Bcl-2, which prevents cytochrome c release from mitochondria [30]. In line with earlier studies, SHH reduced STAT3 activity, increased Bax expression, and decreased Bcl-2 (Figure 7). In addition, SHH induced the upregulation of p21, p27, and p53 and the downregulation of cyclin B1. The STAT3 activator attenuated these effects. However, the caspase inhibitor failed to recover SHH-induced cell death. In addition, the apoptosis markers were not affected by SHH treatment; namely, Caspase 3/7 and 9 activity, PARP cleavage, cytochrome C release, and MMP changes. These results suggest that other mechanisms exist. Interestingly, however, SHH-treated cells showed positive signals in Annexin V/PI and TUNEL staining. Annexin V and TUNEL stainings are used for the monitoring of early and late apoptosis, respectively. SHH-treated macrophages showed M1 phenotypes with short spreading, vacuoles, and flat round shape. A549 cells also showed changed phenotypes with membrane blebbing and numerous vacuoles, like activated macrophages. Membrane damage and DNase release may explain Annexin V and TUNEL staining in the absence of changes in other apoptotic markers in SHH-treated A549 cells (Figure 8). Necrotic cells stained by PI were also seen in the SHH-treated cells. In addition, SHH-treated A549 cells induced IL-1β secretion and caspase-1 activation (Figure 8E). Senescence events and autophagy markers were not consistently detected in SHH-treated A549 cells (Figure 8F).

Pyroptosis and necroptosis are mechanisms of regulated cell death distinct from apoptosis. Lytic cell death requires gasdermin D (GSDMD) for pyroptosis and mixed lineage kinase domain like pseudokinase (MLKL) for necroptosis. Both GSDMD and MLKL result in IL-1β-driven inflammation, which is from caspase-1 activation [35]. Pyroptotic and necroptotic cell death show distinct morphological differences; reduced cell swelling and flattened cytoplasm in pyroptosis, and cell swelling and subsequent osmolysis in necroptosis. However, both cell death mechanisms have in common that they damage the plasma membrane [36,37]. Annexin V staining could not distinguish between apoptosis, pyroptosis, or necroptosis. In this study, SHH-treated cells showed lytic cell death with reduced cell volume, membrane alteration, Caspase-1 activation, and IL-1β secretion. The pyroptotic and necroptotic death pathways are thought to be crosstalked. Macrophages undergoing pyroptosis exhibit both apoptotic and necrotic morphological characteristics [38]. Z-YVAD-FMK and necrostatin-1, which block pyroptosis and necroptosis, inhibited SHH-induced cell death. However, the chemicals were not fully recovered cell death induced by SHH (Figure 8J), indicating that other mechanism may exist in SHH-induced cell death.

Several immunotherapies have been widely applied to cancer to inhibit malignant activity or to activate the immune system against the immune evasion mechanism of cancer [39,40,41]. Most natural immune modulators induce M1 or M2 polarization in one direction, but SHH beneficially affect both M1 and M2 polarization in macrophages for treating cancer. Collectively, our results suggest that SHH may be applied as a therapeutic substance for the activation of the immunocompromised environment in relation to cancer progression. A better understanding of the mechanisms and physiological roles of pyroptosis and necroptosis will be able to explain the mechanism by which SHH cause cancer cell death and anti-cancer immune responses. In addition, the effect of SHH on PD-1 and PD-L1 expression should be investigated in further studies.

## 4. Materials and Methods

### 4.1. Ethical Approval and Chemicals

All experiments were performed with the approval of the Ethics Committee and in accordance with the guidelines of the Institutional Animal Care and Use Committee of Gyeongsang National University (GNU-160811-M0036). Unless otherwise stated, all chemicals were purchased from Sigma-Aldrich (St Louis, MO, USA). Stock solutions of colivelin (378 µM; Tocris Bioscience, Bristol, UK), doxorubicin (2 mg/mL), and lipopolysaccharide (LPS; 5 mg/mL) were prepared in distilled water. The mouse IL-4 (0.1 mg/mL), human IL-4 (0.1 mg/mL), human IL-13 (0.1 mg/mL), and human IFN-γ (0.1 mg/mL), which were purchased from PeproTech (Rocky Hill, NJ, USA), were dissolved in distilled water. Bay 11-7085 (50 mM), necrostatin-1 (20 mg/mL; Cayman Chemical, Ann Arbor, MI, USA), PMA (1 mM), SB203580 (25 mM), S3l-201 (50 mM; Selleckchem, Houston, TX, USA), Z-VAD-FMK (10 mg/mL; InvivoGen, San Diego, CA, USA), and Z-YVAD-FMK (20 mM; Abcam, Cambridge, MA, USA) were dissolved in dimethyl sulfoxide (DMSO). U-46619 (28.53 mM) was prepared in methyl acetate. The solutions were then diluted in culture medium to the working concentration. An equivalent concentration or volume of solvents was added in the control group. The final concentrations of DMSO and methyl acetate were ~0.1% (*v/v*).

### 4.2. Preparation of Sea Hare Hydrolysates

The preparation of SHH was performed as previously described [19]. Briefly, sea hare (*Aplysia kurodai*) was washed, blanched, and minced. Flavourzyme (2%) was directly added to the minced sea hare. The minced sea hare in the enzyme solution was adjusted to pH 6.0 with citric acid powder, incubated at 60 °C for 15 h, boiled at 100 °C for 10 min to inactivate the enzyme reaction, and then filtered with a 200 mesh screen sieve. The filtered solution was concentrated to Brix 50 using a rotary vacuum evaporator (WCR-P6, Daihan Scientific Co., Ltd, Wonju, Korea). Three volumes of ethanol were added to the concentrated hydrolysates. The hydrolysate solution was stirred and left to stand for 12 h until precipitation. The precipitate was freeze-dried, ground into powder, and stored at −70 °C until further analysis. At the time of the experiment, the dried materials were dissolved in distilled water at the indicated concentrations.

### 4.3. Cell Culture and Isolation of Peritoneal Macrophages

The RAW264.7 macrophage cell line derived from Abelson murine leukemia virus-induced tumors was obtained from the American Type Culture Collection (ATCC, Manassas, VA, USA). Human non-small cell lung cancer (NSCLC) cell lines (A549 and HCC-366), human breast cancer cell lines (MCF-7 and MDA-MB-231), and the human monocytic THP-1 cell line were obtained from Korean Cell Line Bank (KCLB, Seoul, Korea). The cells were cultured in Dulbecco’s modified Eagle’s medium (DMEM; Gibco-BRL, Gaithersburg, MD, USA) or RPMI 1640 (Lonza, Walkersville, MD, USA; for THP-1), supplemented with 10% fetal bovine serum (FBS; Gibco-BRL) and 1% penicillin/streptomycin (100 μg/mL), in a humidified atmosphere with 5% CO_2_ at 37 °C. The medium was replaced every 2 days.

Peritoneal macrophages were isolated from C57BL/6 mice (6-week-old, male, *n* = 10) purchased from Koatech Co. (Animal Breeding Center, Pyongtaec, Korea), as previously described [42]. Briefly, 1 mL of 3% Brewer thioglycollate broth (BD Biosciences, Franklin Lakes, NJ, USA) was injected per mouse into the peritoneal cavity. After four days of injection, the peritoneal cells were collected by injecting and aspirating 10 mL PBS from the peritoneum. To evaluate the purity of the isolated macrophages, the peritoneal cells were examined using a flow cytometer, as detailed in Section 4.13.

### 4.4. Macrophage Polarization and Generation of Tumor-Associated Macrophages (TAMs)

THP-1 cells (2 × 10^5^ cells/mL) were seeded in a 6-well plate and cultured for 24 h in RPMI medium. The cultured cells were differentiated into a macrophage-like phenotype by adding 150 nM PMA. After 24 h, the macrophages were polarized into M1 in the presence of 100 ng/mL LPS and 20 ng/mL IFN-γ for 48 h, or into M2 by adding 20 ng/mL IL-4 and 20 ng/mL IL-13 for 72 h. SHH (100 μg/mL) was treated to M0 alone or in combination with M1 or M2 inducers for 48 h or 72 h. The supernatant of each group was harvested just before media change to prepare the conditioned medium. THP-1-differentiated macrophages were co-cultured with A549 cells at a 1:1 ratio for 4 days to generate TAMs.

### 4.5. Cell Viability and Real-time Live Cell Imaging

Cell viability was determined colorimetrically using the 3-(4, 5-dimethylthiazole-2-yl)-2, 5-diphenyl tetrazolium bromide (MTT) reagent (Duchefa, Haarlem, The Netherlands). The MTT assay procedures were performed as previously described [43]. Briefly, cells at the exponential phase were seeded (2 × 10^4^ cells/mL) in a 96-well plate. After treatment with SHH, 10 μL of 5 mg/mL MTT solution was added to each well and incubated for 2 h. The supernatants were then aspirated, the formazan crystals in each well were dissolved in 100 μL of DMSO for 10 min at room temperature, and the plate was read at 570 nm using a microplate reader (Molecular Devices, San Jose, CA, USA). Data are expressed as the optical density or percentage of viable cells compared to the control.

Real-time live cell imaging was performed using Cell Observer (Carl Zeiss, Jena, Germany). Briefly, the cells were seeded at the density of 1.5 × 10^3^ cells/well on Culture-Insert 2-well (ibidi GmbH, Martinsried, Germany) and incubated in a humidified atmosphere with 5% CO_2_ at 37 °C for 48 h. The cells were treated with 100 µg/mL SHH, colivelin was pretreated for 2 h, and then transferred to the Cell Observer. The cells were maintained at 37 °C in 5% CO2. Images were captured at 10 min intervals for 48 h. Cells cultured in the medium without adding SHH were taken as a control.

### 4.6. Live/dead Cell Staining

Live/dead cell staining was performed using calcein-AM (Thermo Fisher Scientific, Eugene, OR, USA) and propidium iodide (PI). Viable cells and disordered areas of dead cell membrane were stained with Calcein-AM and PI and appeared green and red, respectively. A549 cells at the density of 5 × 10^3^ cells/100 μL were cultured on glass-bottomed culture dish (SPL, Pocheon, Korea) for 48 h. The cells were treated with 100 µg/mL of SHH for 24 h, 48 h, or 72 h. The cells were washed twice with free DMEM and then stained with 2 μM calcein-AM and 3 μg/mL PI for 10 min at 37 °C. The stained cells were washed three times and observed using a confocal laser scanning microscope (Olympus, Tokyo, Japan) with filter sets for fluorescein isothiocyanate (FITC) and Texas Red.

### 4.7. Reverse Transcriptase (RT)—Polymerase Chain Reaction (PCR) Analysis

Total RNA from the cells was extracted with Trizol reagent (Thermo Fisher Scientific) according to the manufacturer’s instructions. First strand cDNA was synthesized from the total RNA (3 μg) isolated from the RAW264.7 cells, the mouse peritoneal and human THP-1-differentiated macrophages, and the TAMs using oligo dT (DiaStar RT Kit, SolGent, Daejeon, Korea). The first-strand cDNA was quantified using a spectrophotometer (NanoDrop^®^ ND-1000, NanoDrop Technologies, Wilmington, DE, USA). The quantified cDNA was used as a template for PCR amplification with G-*Taq* polymerase (Cosmogenetech, Seoul, Korea). PCR assay was performed with specific primers for M1 and M2 polarization markers. The specific primer sequences are listed in Table 1. The PCR steps included initial denaturation at 94 °C for 5 min, then 32 cycles at 94 °C for 30 s, 55 °C to 60 °C (see Table 1) for 30 sec, 72 °C for 30 sec, and a final extension step at 72 °C for 10 min. The amplified PCR products were separated in 1.5% agarose gel stained with ethidium bromide. The bands obtained by RT-PCR were extracted and directly sequenced with an ABI PRISM^®^ 3100-Avant Genetic Analyzer (Applied Biosystems, Carlsbad, CA, USA).

### 4.8. Western Blot Analysis

Peritoneal and THP-1-differentiated macrophage cells and A549 cells were lysed in RIPA buffer (25 mM Tris-HCl; pH 7.4, 150 mM NaCl, 1% NP-40, 1% sodium deoxycholate, 0.1% SDS) containing protease and phosphatase inhibitors. The procedures of Western blot analysis were as described previously [43]. Briefly, the cell lysates were incubated for 30 min on ice with intermittent vortexing and were clarified by centrifugation at 15,000 rpm (22,250× *g*; Micro 17TR, Hanil, Incheon, Korea) for 20 min at 4 °C. After centrifugation, the supernatant was separated and stored at −70 °C until use. Protein concentration was quantified using a Pierce bicinchoninic acid (BCA) Protein Assay Kit (Thermo Fisher Scientific, Waltham, MA, USA). Equal amounts (35 μg) of total protein were analyzed among experimenal groups. Equal volumes of the supernatant and 2× sodium dodecyl sulfate (SDS) sample buffer were mixed, loaded on 10% SDS-polyacrylamide gel, and separated by electrophoresis for 120 min at 120 V. Then, the gel was transferred to a polyvinylidene difluoride membrane (Millipore, Billerica, MA, USA) for 1 h at 100 V using a wet transfer system (Bio-Rad, Hercules, CA, USA). The membranes were blocked with 5% fat-free dry milk and then incubated with anti-phospho-p38 MAPK pThr180+Tyr182 (1:1000 dilution; Thermo Fisher Scientific), anti-p38 MAPK (1:1000 dilution; Thermo Fisher Scientific), anti-phospho-STAT3 (Tyr705, 1:2000 dilution; Cell Signaling, Danvers, MA, USA), anti-STAT3 (1:1000 dilution; Cell signaling), anti-caspase-1 (1:1000 dilution; Abcam), anti-p21 (1:1000 dilution; Santa Cruz Biotechnology, Dallas, TX, USA), anti-p27 (1:1000 dilution; Santa Cruz Biotechnology), anti-p53 (1:1000 dilution; Santa Cruz Biotechnology), anti-Cyclin B1 (1:1000 dilution; Cell signaling) or anti-β-actin (1:5000 dilution). The primary antibody incubation was followed by incubation with a secondary peroxidase conjugated anti-rabbit or anti-mouse antibody at 1:5000 (Assay designs, Ann Arbor, MI, USA). Immuno-positive bands were visualized by enhanced chemiluminescence reagent (Dogen, Seoul, Korea) according to the manufacturer’s instructions.

### 4.9. Phagocytosis Assay

The phagocytic ability of macrophages was analyzed using a phagocytosis assay kit (Cayman Chemical) according to a previously described method [18]. Briefly, 5 × 10^4^ cells/well RAW 264.7 cells were cultured in a 96-well black-bottomed plate. The cultured cells were stimulated with LPS (1 μg/mL) or SHH (1, 10, and 100 μg/mL). Then, 10 μL of the latex beads rabbit IgG FITC solution was added and incubated for 2 h. The cells were washed three times with the assay buffer. The washed cells were analyzed for fluorescence intensity using a fluorescence microplate reader (Tecan, Maennendorf, Switzerland). The phagocytic ability of macrophages was also analyzed through co-culture with A549 cells, which were transfected with green fluorescent protein (GFP) in pcDNA3.1 using LipofectAMINE2000 (Thermo Fisher Scientific) and Opti-MEM I (Thermo Fisher Scientific). The A549 cells were added to RAW264.7 cells cultured in a 35 mm cell imaging dish with a glass coverslip bottom, and SHH was added to the dish. Green fluorescence from cells expressing GFP was detected with a confocal laser scanning microscope equipped with a fluorescence system (IX70 Fluoview, Olympus).

### 4.10. Migration Assay

For wound-healing (two-dimensional (2D)) migration assays, cells were seeded onto a 24-well culture dish at a density of 2 × 10^5^ cells/well. Cells were scratched with a sterile pipette tip, washed with PBS, and incubated in fresh medium for 24 h. Another assay was performed using Culture-Insert 2-well (ibidi GmbH) according to the manufacturer’s protocol. Briefly, A549 cell suspension at the density of 66 × 10^4^ cells/mL (70 µL) was seeded into each well, and the cells were incubated for 24 h, then washed three times with free medium. After washing, the cells were captured as the initial images at 0 h. Macrophage conditioned media were added to the cells and incubated for 12 or 24 h at 37 °C. Photomicrographs were taken after incubation for the periods indicated. Relative cell migration distance (wound closure) was determined by measuring the wound width on the monolayer using AxioVision 4.5 software under a microscope (Axiovert 40C, Carl Zeiss MicroImaging, Göttingen, Germany) and by dividing this value from the initial value (i.e., the initial wound width at 0 h). The data acquired from the three scratches on each well were converted to the percentage of wound closure at a given time.

The transwell (three-dimensional (3D)) migration assay was performed using the CytoSelect^™^ Cell Migration Assay Kit containing polycarbonate membrane inserts (0.8 μm pore membrane; Cell Biolabs, Inc., CA, USA) according to the manufacturer’s instructions. The cell suspension (2 × 10^4^ cells/300 μL) in macrophage conditioned media was added to the upper chamber of each insert. The lower chamber contained medium with 10% FBS to allow cell migration toward the lower face of the transwell culture inserts. Cells were incubated for 24 h at 37 °C in a 95% air—5% CO_2_ gas mixture. Non-migrating cells on the inner side of the transwell culture inserts were gently removed with a cotton-tipped swab. Migrated cells remaining on the bottom surface were stained with a cell-staining solution (0.4% crystal violet) for 10 min at room temperature. Photomicrographs of five individual fields per insert were taken using a microscope (Axiovert 40C).

### 4.11. Colony Formation Assay

A549 cells at the density of 5 × 10^3^/well were seeded in a 6-well plate and grown in DMEM medium supplemented with 10% FBS and 1% penicillin/streptomycin. After 24 h, different concentrations of SHH were added to the cells for 48 h. The medium was replaced with a fresh medium every 2 days for 14 days. On the last day, the colonies were fixed in methanol for 10 min. The methanol was removed and the colonies were air-dried for 30 min until white in color. After that, the colonies were stained with 0.1% crystal violet solution for 30 min. The stain solution was removed, and the colonies were washed five times with distilled water. Finally, the colonies were air-dried and captured.

### 4.12. Tumor Spheroid Assay

The preparation of 1.5% agarose and spheroid formation was followed as previously described [44]. Agarose/DMEM mixture (1.5%) was added to a 96-well plate to generate agarose-coated well. The prepared A549 single cell suspension was added into each well of the agarose-coated plate (2.5 × 10^3^/200 μL/well). The cell suspensions were incubated for 4 days in a humidified atmosphere with 5% CO_2_ at 37 °C. After 4 days, the supernatant of 100 μL was replaced with SHH-supplemented media, and then cultured for 10 days. Finally, the spheroid images were captured using AxioVision 4.5 software under a microscope (Axiovert 40C).

### 4.13. Flow Cytometric Analysis and Staining

Flow cytometric analysis was performed using a BD FACSAria II flow cytometer (BD Biosciences, Franklin Lakes, NJ, USA) with FACSDiva software version 6.1.2 (BD Biosciences). The analysis of mouse peritoneal macrophages was performed using the following antibody: PE-conjugated anti-mouse CD14 (Biolegend, San Diego, CA, USA). The cells and 1 μL of anti-CD14 PE antibody were incubated for 40 min at 4 °C in the dark. The cells were then washed twice to remove unbound antibodies using washing buffer (PBS with 1% BSA). Subsequently, the cells were analyzed by flow cytometry with fixation (4% paraformaldehyde).

A549 cells were cultured at a density of 8 × 10^4^ cells/well in a 60 mm dish for 48 h. The cells were treated with 100 μg/mL SHH for 48 h, trypsinized, fixed with cold 70% ethanol, and incubated for 1 h at −20 °C. The cells were pelleted and resuspended in 1 mL PBS containing PI (1 mg/mL) and RNase A (1 mg/mL). Following incubation at 25 °C for 30 min, cell cycle and apoptosis (subG1) were determined by a flow cytometer (FACscan, BD Biosciences) with CXP 2.2 software (Beckman Coulter, Brea, CA, USA).

### 4.14. Cellular Senescence Assay

Cellular senescence assay was performed according to the manufacturer’s protocol (BiMilpitas, CA, USA). Briefly, A549 cells at density of 9 × 10^3^ cells/mL were seeded in a 24-well plate and incubated at 37 °C and 5% CO_2_ for 24 h, and the cells were further incubated in the presence of SHH treatment for 48 h. The cells were washed once with 1mL of 1 × PBS, and were fixed with 0.5 mL of a fixative solution for 15 min at room temperature. The fixed cells were washed twice with 1 mL of 1× PBS. Finally, a staining solution mixture (470 µL of staining solution + 5 µL of staining supplement + 25 µL of 20 mg/mL X-gal in DMF) was added to each well, and the plate was incubated overnight at 37 °C. The images were captured using AxioVision 4.5 software under a microscope (Axiovert 40C).

### 4.15. Caspase 3/7 Assay

A549 cells at a density of 3.5 × 10^4^/mL were seeded in a 96-well black-bottomed plate. The cells were grown in DMEM supplemented with 10% FBS and 1% penicillin/streptomycin for 48 h. The cells were treated with 100 µg/mL of SHH for 48 h. To detect caspase-3 and -7 activity in the cell-based assay, caspase-Glo^®^ 3/7 reagent (Caspase-Glo^®^ 3/7 substrate + Caspase-Glo^®^ buffer; Promega, Madison, WI, USA) was added to the cells in the medium (100 μL of medium + 100 μL of reagent), mixed by orbital shaking (300–500 rpm) for 30 sec, and incubated for 1 h at room temperature. The caspase activity was analyzed by measuring the luminescence of each sample in the GloMax^®^ microplate reader (Promega).

### 4.16. TUNEL Staining

TUNEL assay was performed using the DeadEnd^™^ Fluorometric TUNEL System (Promega) according to the manufacturer’s protocol. Briefly, cells grown on poly-L-lysine-coated cover glass were fixed in 4% paraformaldehyde in PBS for 25 min at 4 °C, washed twice in PBS, and permeabilized in 0.2% Triton X-100 in PBS for 5 min. After the two washes in PBS, the cells were equilibrated in an equilibration buffer for 10 min. The cells were labeled with TdT reaction mix and incubated for 60 min at 37 °C in a dark humidified chamber. The reaction was stopped with 2× saline sodium citrate (SSC) solution, followed by washing three time in PBS. Counter staining was carried out by incubation with 2 μg/mL DAPI for 10 min at room temperature in the dark. TUNEL-positive green fluorescent cells were observed using a confocal laser scanning microscope (Olympus).

### 4.17. Annexin V/PI Staining

Annexin V staining was performed using the Annexin-V-FLUOS staing kit (Roche, Mannheim, Germany) according to the manufacturer’s protocol. Briefly, cells grown on a glass-bottomed culture dish (SPL) were labeled with Annexin-V-Fluos labeling reagent and PI, and then incubated for 15 min at room temperature in the dark. Following incubation, the cells were analyzed using a confocal laser scanning microscope (Olympus).

### 4.18. Caspase-1 Assay

Active caspase-1 was detected using the caspase-1 staining kit (Abcam) according to the manufacturer’s protocol. Briefly, cells grown on poly-L-lysine-coated cover glass were treated with chemicals for 48 h and washed twice with a washing buffer. The FAM-YVAD-FMK was added to the cells in 150 μL of washing buffer at a 1:150 ratio, and the cells were incubated for 60 min at room temperature with 0.3 µL of 500X Hoechst solution. The stained cells were washed and were mounted with permount mounting medium (Fisher Chemical, Geel, Belgium), and then analyzed using a confocal laser scanning microscope (Olympus).

### 4.19. IL-1β Immunoassay

The level of IL-1β in the medium was measured using an enzyme-linked immunosorbent assay (ELISA) kit (R&D Systems, Minneapolis, MN, USA) according to the manufacturer’s protocol. Biefly, cells (5 × 10^5^ cells/mL) were cultured in a 60 mm culture dish, incubated overnight, and stimulated with SHH (100 μg/mL) for 48 h. Cell supernatants were then collected. Cell supernatants, 50 μL of assay diluent, and the standard were added to the a 96-well plate, which was pre-coated with anti-human IL-1β antibody. The plates were covered with an adhesive strip, incubated for 2 h at room temperature, and washed three times with wash buffer. Then, 100 μL of human IL-1β conjugate was added, incubated at room temperature for 2 h, and washed three times. The reaction was quenched by the addition of 100 μL stop solution, and the absorbance of the plates was read at 450 nm with a microplate reader (Molecular Devices, Sunnyvale, CA, USA).

### 4.20. Data Analysis and Statistical Analysis

A LAS-4000 (Fujifilm Corp, Tokyo, Japan) and MaXidoc Gel Imaging System (Daihan Scientific, Wonju, Korea) were used to capture images of the Western blots and agarose gel, respectively. The bands were quantified by ImageJ software (version 1.49, National Institute of Health, Bethesda, MD, USA). Data are presented as the mean ± standard deviation (SD). The one-way ANOVA/Bonferroni test or the Kruskal-Wallis/Mann-Whitney test was chosen after the normality test to analyze the differences among groups (OriginPro2020, OriginLab Corp. Northampton, MA, USA). Significance was set at *p* < 0.05.

## 5. Conclusions

In conclusion, our results demonstrate that SHH induce M1 polarization and decrease M2 polarization in mouse and human macrophages. SHH-induced M2 suppression and anticancer effect on NSCLC cells (A549 and HCC-366) were mediated by STAT3 inhibition. SHH-induced STAT3 inhibition caused non-apototic cell death, similary pyroptosis and necroptosis. Our findings suggest that SHH could be used as a potential therapeutic agent for NSCLC.

## Figures and Tables

**Table 1:** Primer sequences used for PCR.

Gene Name	GenBank Acc. No.	Primer Sequences (5′–3′)	Expected Size (bp)	Species	Annealing Temp. (°C)
*Nos2* (iNOS)	NM_010927	Sense: TTGACGCTCGGAACTGTAGCA	492	Mouse	57
		Antisense: TGCCCATGTACCAACCATTGA			
*TNF* (TNF-α)	NM_013693.3	Sense: CAGCCTCTTCTCTTCTCATTCCTGC	339	Mouse	57
		Antisense: TGTCCCTTGAAGAGAGAACCTG			
*Arg1* (Arg-1)	NM_007482.3	Sense: CAGAAGAATGGAAGAGTCAG	249	Mouse	55
		Antisense: CAGATATGCAGGGAGTCACC			
*Chil3l3* (Ym1)	NM_009892.3	Sense: TGGAATTGGTGCCCCTACAA	344	Mouse	55
		Antisense: AACTTGCACTGTGTATATTG			
*GAPDH*	GU214026	Sense: CTA AAG GGC ATC CTG GGC	201	Mouse	55
		Antisense: TTA CTC CTT GGA GGC CAT			
*fibronectin 1 (FN1)*	NM_212482.3	Sense: CGAGCCCTGAGGATGGAATC	423	Human	60
		Antisense: CAACTCCCTGAGCTGGTCTG			
*CD209*	NM_001144897.1	Sense: CTAACTCCCAGCGGAACTGG	336	Human	60
		Antisense: CTTCATCCCTGGAGCAGGAG			
*MRC1 (CD206)*	NM_002438.3	Sense: TGGAGGGAATCTGGTCTCCAT	433	Human	60
		Antisense: CTTGCAGTATGTCTCCGCTTC			
*IL10*	NM_000572.2	Sense: GCCTTCAGCAGAGTGAAGACT	253	Human	60
		Antisense: GAAGAAATCGATGACAGCGCC			
*CCL18*	NM_002988.3	Sense: CTGTGCTGACCCCAATAAGA	341	Human	58
		Antisense: CGAAGAGTTGAAGGGAAAGGG			
*IL1β*	NM_000576.2	Sense: GAGCTCGCCAGTGAAATGATG	340	Human	58
		Antisense: CAGGTGCATCGTGCACATAAG			
*TNF-α*	NM_000594.4	Sense: AGTGACAAGCCTGTAGCCCAT	434	Human	60
		Antisense: CCAAAGTAGACCTGCCCAGAC			
*IL-6*	NM_000600.5	Sense: AACCTTCCAAAGATGGCTGAA	160	Human	60
		Antisense: GCAGGAACTCCTTAAAGCTGC			
*Arg1* (Arg-1)	NM_001244438.1	Sense: CAAGTCCAGAACCATAGGGA	242	Human	50
		Antisense: CTTTTCCCACAGACCTTGGA			
*GAPDH*	NM_002046.7	Sense: CCCATGTTCGTCATGGGTGT	145	Human	55
		Antisense: TGGTCATGAGTCCTTCCACGATA			
